# Pruritus in Hemodialysis Patients in a Sub‐Saharan African Country: Benin—Associated Skin Conditions and Other Factors in 2023 in Three Hemodialysis Centers

**DOI:** 10.1155/drp/4482167

**Published:** 2026-03-09

**Authors:** Dégboé Bérénice, Tounouga Dahlia Noelle, Gbénou Fabrice Mahouéna, Lossou Eric, Hazoumè Rodrigue, Vigan Jacques, Atadokpèdé Félix

**Affiliations:** ^1^ Department of Dermatology and Venereology, Faculty of Health Sciences, Hubert Koutoukou Maga National Teaching Hospital Center (CNHU-HKM) of Cotonou, University of Abomey-Calavi, Cotonou, Benin, uac.bj; ^2^ Dialysis Unit UNIDIAL, Cotonou, Benin; ^3^ Dialysis Unit LONGUE VIE, Abomey-Calavi, Benin; ^4^ Department of Nephrology and Hemodialysis, Faculty of Health Sciences, Hubert Koutoukou Maga National Teaching Hospital Center (CNHU-HKM) of Cotonou, University of Abomey-Calavi, Cotonou, Benin, uac.bj

**Keywords:** Beau’s lines, Benin, chronic kidney disease, hemodialysis, pruritus, severe xeroderma

## Abstract

**Introduction:**

Pruritus is a frequent and debilitating complication in hemodialysis patients. Its pathophysiology remains poorly understood. This study aimed to describe the factors associated with pruritus in three hemodialysis centers in 2023.

**Methods:**

This was a prospective, descriptive, and analytical cross‐sectional study. It included 245 patients aged 18 years and older, undergoing hemodialysis for at least three months, hemodynamically stable, and who had provided informed consent. Pruritus intensity was assessed using the Itch‐Numeric Rating Scale (Itch‐NRS). Statistical analysis was performed using Stata 17, with a significance level set at *p* < 0.05.

**Results:**

The prevalence of pruritus among hemodialysis patients was 40.41%. The mean age of participants was 52.75 years, with a sex ratio of 1.78. Pruritus was most often mild to moderate (90.91%), generalized (48.48%), or diffuse (51.52%) and had been present for at least 1 year in 67.67% of cases. The most frequently associated dermatoses were xeroderma (88.88%), nail abnormalities (77.14%), and diffuse hyperpigmentation (27.27%).

Univariate analysis identified protective factors such as vitamin D supplementation, absence of anuria, absence of diffuse skin hyperpigmentation, absence of alopecia, and absence of nail abnormalities (*p* = 0.003–0.042; OR = 0.17–0.58). Multivariate analysis showed that prolonged duration of hemodialysis, the use of an arteriovenous fistula, severe xeroderma, and the presence of Beau’s lines (*p* ≤ 0.039) increased the risk of pruritus in hemodialysis patients. Conversely, the use of oral antidiabetic agents was significantly associated with a reduced risk of pruritus (*p* = 0.003; OR = 0.25).

**Conclusion:**

Pruritus is a common and chronic condition in hemodialysis patients, adversely affecting their quality of life. The main risk factors identified were severe xeroderma, prolonged duration of hemodialysis, use of an arteriovenous fistula, and the presence of Beau’s lines, whereas the use of oral antidiabetic medications appeared to be protective. A better understanding of the pathophysiology of pruritus in hemodialysis patients could help improve its management and enhance patient comfort.

## 1. Introduction

Among the various dermatological manifestations observed in hemodialysis patients, chronic pruritus stands out due to both its high frequency and its particularly debilitating nature [1–5]. It significantly impairs quality of life, contributes to sleep disturbances, and has substantial psychosocial consequences [6, 7]. Its complex and still poorly understood pathophysiology makes management challenging, and available treatments—nonstandardized and often limited—provide only moderate efficacy [8–10].

Studies conducted in sub‐Saharan Africa and in other regions have consistently reported a high prevalence of pruritus among hemodialysis patients [1–3, 11, 12]. Other authors have noted that in resource‐limited settings, pruritus in hemodialysis patients is underdiagnosed and inadequately managed [2, 12]. Despite its frequency and the higher burden of morbidity within this population, it remains insufficiently studied. It is therefore essential to conduct research on black skin in sub‐Saharan Africa in order to reduce this disparity and to ensure better understanding of the pathophysiology, as well as improved validity, comparability, and applicability of data to diverse populations.

In this context, this multicenter study was conducted in Benin to describe the key characteristics of pruritus in hemodialysis patients and to identify associated factors.

## 2. Methods

This was a multicenter, cross‐sectional, prospective, and analytical study conducted from 25 September to 25 December 2023 in three hemodialysis centers in Benin: the University Clinic of Nephrology and Hemodialysis (CUNH) and two private facilities (Unidial and Longue Vie) located in two different cities.

Patients aged 18 years and older, with chronic kidney disease (CKD), and undergoing hemodialysis for at least 3 months were included. During the interview, we ensured that the chronological characteristics supported a causal link with hemodialysis—namely, that the onset of symptoms coincided with the start of hemodialysis sessions (within at least 3 months) and that the temporal pattern of pruritus progression supported hemodialysis as the likely cause.

Pruritus intensity was assessed using the Itch‐Numeric Rating Scale (Itch‐NRS). The Itch‐NRS is a unidimensional tool designed to evaluate the self‐reported severity of the most intense pruritus experienced each day. Patients were asked to rate pruritus intensity based on the highest level of itching perceived during the previous 24 h, using an 11‐point numerical scale ranging from 0 (no pruritus) to 10. Scores of 1–3 were considered mild pruritus, 4–5 moderate, 6–7 severe, and 8–10 very severe [13].

Xerosis severity was assessed using the SRRC score (scaling, roughness, redness, and fissures). The SRRC score is the sum of the four elementary lesion scores, each rated on a scale from 0 to 4. The total score thus ranges from 0 (normal skin) to 16 (extreme xerosis). Dryness was considered mild for SRRC scores of 1–4, moderate for 5–8, severe for 9–12, and extreme for 13–16 [14].

Only hemodynamically stable and consenting patients were included. Patients undergoing hemodialysis for less than 3 months or presenting with pruritus due to causes unrelated to CKD—such as pruritus associated with an identifiable dermatologic condition or another internal disease—were not included.

Eligible patients were recruited during their hemodialysis sessions. After obtaining written informed consent, each participant underwent an individual interview and a complete physical examination performed by a dermatologist. Laboratory data were extracted from dialysis records.

All data were entered into KoboCollect and analyzed using STATA IC 17. Quantitative variables were described using means and standard deviations or medians according to their distribution; qualitative variables were expressed as percentages. Associations were assessed using Pearson’s chi‐square test or Fisher’s exact test when appropriate. A backward logistic regression model was used for multivariate analysis. Variables with *p* ≤ 0.20 in univariate analysis were included in the final model, and only those with *p* ≤ 0.05 were retained. Model performance was evaluated using the area under the receiver operating characteristic (ROC) curve.

The study was conducted in accordance with the principles of the Ethics Committee of the Faculty of Health Sciences of Cotonou and applicable ethical guidelines. Authorization was obtained from the administrations of the participating centers. Each participant received an information sheet, and written informed consent was required prior to questionnaire administration and physical examination. Confidentiality, anonymity, and participants’ rights were upheld throughout the study.

## 3. Results

**FIGURE 1:**
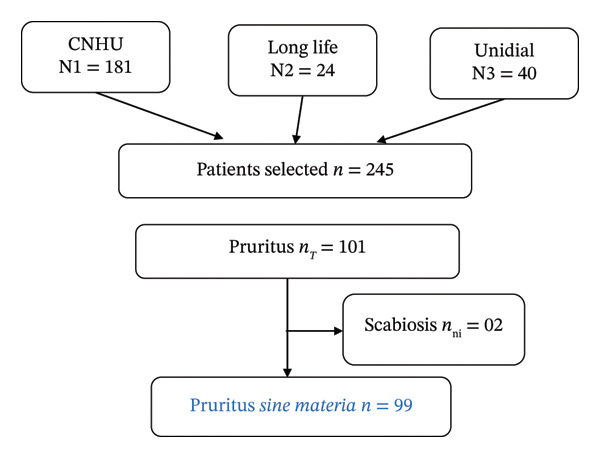
Patient flow diagram.

A total of 245 patients were enrolled across the three centers during the study period, among whom 99 presented with pruritus, corresponding to a hospital prevalence of 40.41% (95% CI: 34.3–46.5) (Figure 1).

The mean age of hemodialysis patients was 52.75 ± 11.69 years (range: 23–84 years). The study population was predominantly male (sex ratio: 1.78). Arterial hypertension was reported in all patients, while 20.20% had diabetes.

Pruritus was mild in 51.52% of cases and moderate in 39.39%. It was generalized (48.48%) or diffuse (51.52%) and had been present for at least 1 year in 67.67% of cases. The main triggering factors identified were hemodialysis sessions (64.65%), xerosis (24.24%), and ambient heat (19.19%). The most effective relieving factors were extensive rinsing of the extracorporeal circuit with normal saline before dialysis (31.31%) and the use of antihistamines (21.21%).

**FIGURE 2:**
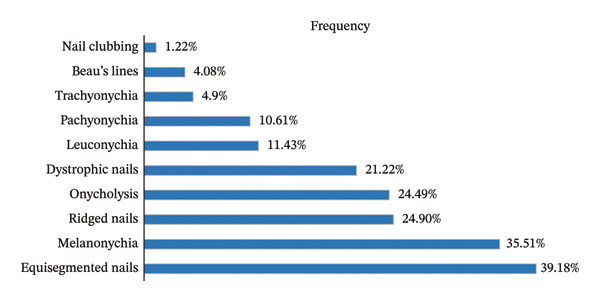
Distribution of included patients by nail changes.

Dermatological findings primarily included xeroderma (88.88%), followed by diffuse hyperpigmentation (27.27%). Nail changes were present in 77.14% of patients: equisegmented nails (39.18%), melanonychia (35.51%), nail ridges (24.90%), onycholysis (24.49%), and nail dystrophy (21.22%). Beau’s lines were identified in 4.08% of patients. These findings are illustrated in Figure 2.

### 3.1. Univariate Analysis Identified Several Factors Associated With Pruritus

**Protective factors** included vitamin D supplementation, absence of anuria, absence of diffuse skin hyperpigmentation, absence of alopecia, absence of nail abnormalities, and a slight increase in CRP, with *p* values ranging from 0.003 to 0.042 and odds ratios (ORs) from 0.17 to 0.58.

**Risk factors** included a dialysis duration ≥ 12 months, use of an arteriovenous fistula (AVF), aspirin intake, severe xerosis, and the presence of Beau’s lines, with *p* values between 0.0001 and 0.047 and ORs ranging from 2.51 to 14.5 (Table 1).

**TABLE 1:** Factors associated with pruritus—univariate analysis.

	**Pruritus n (%)**		**OR**	**[CI (95%)]**	**p** **value**
	**Yes**	**No**			

*Duration of hemodialysis (months)*					**0.0008**
[3–6]	01 (14.29)	06 (85.71)	1	—	—
[6–12]	03 (13.64)	19 (86.36)	0.94	[0.08–10.89]	0.965
[12–60]	18 (29.51)	43 (70.49)	2.51	[0.28–22.38]	0.409
[60–120]	39 (46.99)	44 (53.01)	5.31	[0.61–46.13]	0.130
≥ 120	38 (52.78)	34 (47.22)	6.70	[0.76–58.55]	0.085

*Type of vascular access*					**0.0001**
Simple catheter	02 (08.00)	23 (92.00)	1	—	—
Tunneled catheter	00 (00.00)	11 (100)	—	—	—
Arteriovenous fistula	97 (46.41)	112 (53.59)	9.95	[2.28–43.32]	**0.002**

*Anuria*					**0.001**
Yes	89 (45.18)	108 (54.82)	1	—	—
No	10 (20.83)	38 (79.17)	0.319	[0.15–0.67]	**0.003**

*Vitamin D*					**0.039**
No	60 (46.51)	69 (53.49)	1	—	—
Yes	39 (33.62)	77 (66.38)	0.58	[0.34–0.97]	**0.041**

*Aspirin intake*				**0.034**	
No	06 (22.22)	21 (77.78)	1		—
Yes	93 (42.66)	125 (57.34)	2.60	[1.01–6.70]	**0.047**

*Xeroderma*					**0.0001**
Mild	22 (30.99)	49 (69.01)	1.42	[0.61–3.32]	0.407
Moderate	27 (40.91)	39 (59.09)	2.20	[0.95–5.08]	0.064
Severe	39 (62.90)	23 (37.10)	5.39	[2.30–12.63]	**0.0001**

*Note:* The values highlighted in bold in the table correspond to the *p* values that reached the threshold of statistical significance in the statistical analysis.

Abbreviations: CI, confidence interval; OR, odds ratio.

Past medical history—including arterial hypertension (*n* = 243), diabetes (*n* = 52), the underlying nephropathy leading to hemodialysis (vascular: *n* = 181; diabetic: *n* = 52; polycystic kidney disease: *n* = 9; and sickle cell disease: *n* = 1), pruritic dermatoses (*n* = 22), atopy (*n* = 40), hepatitis B (*n* = 15), hepatitis C (*n* = 19), HIV infection (*n* = 10), and iron deficiency (*n* = 5)—was not associated with hemodialysis‐related pruritus (0.119 < *p* < 0.987). Various hematologic abnormalities were identified, but none were significantly associated with pruritus in hemodialysis patients (0.058 < *p* < 0.923). Protein abnormalities (hypoproteinemia, *n* = 16, and hyperproteinemia, *n* = 3) and calcium disturbances (hypocalcemia, *n* = 40) were also not significantly associated (0.062 < *p* < 0.660).

Lifestyle factors—including the use of scented cosmetics (*n* = 148), skin‐lightening products (*n* = 31), antiseptics (*n* = 57), detergents (*n* = 183), nail polish (*n* = 62), herbal medicine (*n* = 110), and absence of deworming (*n* = 72)—were not associated with pruritus (0.087 < *p* < 0.837).

Multivariate logistic regression analysis showed that use of an AVF, severe xerosis, and the presence of Beau’s lines were significantly associated with pruritus (*p* ≤ 0.039). Conversely, the use of oral antidiabetic medications was associated with a reduced risk of pruritus (*p* = 0.003; OR = 0.25) (Table 2).

**TABLE 2:** Multivariate analysis of factors associated with pruritus.

	Adjusted OR	[CI (95%)]	*p* value
Vascular access via AVF^∗^	7.71	[1.46–40.69]	**0.016**
Medications			**0.027**
Oral antidiabetic	0.25	[0.10–0.62]	**0.003**
Vitamin D	0.50	[0.25–1.00]	0.053
Severe xeroderma	3.03	[1.33–6.89]	**0.008**
Nail abnormalities			
Beau’s lines	10.87	[1.12–105.06]	**0.039**

*Note:* The values highlighted in bold in the table correspond to the *p* values that reached the threshold of statistical significance in the statistical analysis.

^∗^OR: odds ratio; CI: confidence interval.

## 4. Discussion

Few recent multicenter studies have investigated pruritus associated with CKD in West Africa. This study addresses an important epidemiological gap in low‐ and middle‐income countries, where dermatological manifestations of CKD are often underreported and undertreated, despite evidence showing higher prevalence rates and morbidity burdens in these populations.

The prevalence of pruritus observed in this study was 40.41%, which is consistent with findings reported in similar settings such as Burkina Faso (45.3%), Indonesia (40.3%), and Jordan (48.1%) [12, 15, 16]. This prevalence highlights the high frequency of pruritus among hemodialysis patients, a multifactorial condition that is often underdiagnosed. However, our results differ from those of other studies reporting higher rates (up to 83.7%) or, conversely, lower rates [7, 17–19]. These discrepancies may be explained by variations in diagnostic approaches, ranging from standardized questionnaires to subjective patient self‐assessments. Moreover, limited access to dermatologic care in certain regions may lead to an underestimation of pruritus prevalence due to less accurate diagnosis.

The mean age of participants was 52.75 ± 11.69 years. This result is comparable to that reported in Nigeria [2] but slightly higher than that observed in Burkina Faso and Jordan [12, 16], both low‐ and middle‐income countries. Conversely, studies conducted in South Korea and China have reported higher mean ages, likely related to increased life expectancy associated with stronger healthcare systems [5, 20]. In resource‐limited countries, restricted access to healthcare and late diagnosis of CKD often contribute to the onset of end‐stage renal disease (ESRD) at a younger age [21, 22].

The male predominance observed in our study (64.08%, sex ratio = 1.78) is consistent with data from the literature [12, 16, 19, 23, 24], suggesting a possible role of sex hormones. Testosterone promotes podocyte apoptosis and renal fibrosis, whereas estradiol exerts a protective effect [25, 26]. These hormonal mechanisms may explain the greater susceptibility of male patients to more rapidly developing severe renal impairment and, consequently, pruritus associated with hemodialysis at the terminal stage of disease [27, 28].

Previous studies have demonstrated a strong association between hemodialysis sessions and pruritus [29–31]. The onset or exacerbation of pruritus during dialysis may reflect hypersensitivity to dialyzer components (tubing, catheters, and membranes) or incomplete removal of uremic toxins [12, 19, 32]. This hypothesis is supported by the observation that some patients reported relief when the dialyzer was thoroughly rinsed before each session—a simple, low‐cost strategy particularly suitable for resource‐limited settings. However, Masmoudi et al. reported that some patients experienced relief during dialysis itself, likely due to partial clearance of circulating pruritogenic substances [32].

Dermatological manifestations were dominated by xerosis, consistent with findings from other studies [3, 12, 18, 33]. Variations in the reported frequency of xerosis may be linked to heterogeneity in etiological factors, individual cosmetic habits, and regional climatic conditions. Cutaneous hyperpigmentation, when present, is thought to result from increased melanin accumulation in the basal layer and superficial dermis, secondary to impaired renal clearance of *β*‐melanocyte‐stimulating hormone (β‐MSH) by the kidneys and dialysis membranes [12, 19]. In our study, hyperpigmentation was observed in a smaller proportion of patients. Low proportions have also been reported in other populations with darker skin phototypes [1–3, 12]. These observations contrast with findings from populations with lighter phototypes where higher rates have been described [19] and even more markedly among fair‐skinned individuals [18, 30, 33]. The lower detection rates of hyperpigmentation in darker skin may reflect underestimation, likely due to the subtle contrast between baseline pigmentation and pathological changes, making such variations more difficult to identify.

Nail changes (including half‐and‐half nails, melanonychia, and dystrophic changes) were present in 77.14% of patients. Although nonspecific, these alterations reflect underlying metabolic and nutritional imbalances frequently observed in hemodialysis populations [12, 17, 18]. In the series by Deshmukh et al., Beau’s lines represented the most common nail abnormality, affecting 28.57% of patients, whereas Al Thnaibat et al. reported a frequency similar to ours [16, 19]. In our study, Beau’s lines appeared to be a risk factor for pruritus among hemodialysis patients. This finding should, however, be interpreted with caution. We believe that there is no direct causal relationship between Beau’s lines and pruritus; rather, Beau’s lines may be a clinical marker associated with xerosis, which itself is strongly linked to pruritus in hemodialysis patients.

Multivariate analysis showed that patients with severe xerosis had a 5.39‐fold increased risk of developing pruritus, confirming its major role in the underlying pathophysiology. Several studies have similarly demonstrated that patients with severe xerosis are significantly more likely to experience intense pruritus [15, 24, 33–36].

The use of oral antidiabetic agents was identified as a protective factor against pruritus (*p* = 0.003). Oral antidiabetic medications, such as sodium‐glucose cotransporter 2 (SGLT2) inhibitors (gliflozins) and glucagon‐like peptide‐1 (GLP‐1) receptor agonists, have been shown to reduce systemic inflammation and oxidative stress—two key mechanisms in uremic pruritus [37, 38]. However, the protective role observed in our study should be interpreted cautiously, as this association may be confounded by diabetes itself, glycemic control, or residual renal function. Ultimately, better glycemic control may contribute to improved skin barrier integrity, thereby reducing xerosis and, consequently, pruritus.

Longer duration of hemodialysis also emerged as a risk factor. This may be related to cumulative retention of pruritogenic uremic toxins (such as β2‐microglobulin and parathyroid hormone) and reduced cutaneous clearance resulting from prolonged exposure to suboptimal dialysis efficiency. Such conditions may foster a chronic inflammatory state that exacerbates pruritus, supporting a dose–response relationship [8, 34–39].

The use of an AVF was also significantly associated with pruritus (97.98% of affected patients). This association may be explained by the formation of microthrombi, vascular alterations, and chronic inflammatory responses that could indirectly contribute to the onset of pruritus [40].

### 4.1. Limitations of the Study

This study presents several limitations, including its urban setting and geographical restriction, which may limit the generalizability of our findings to the entire Beninese population. Hemodialysis centers are concentrated in three major cities, with Cotonou—the economic capital and study site—hosting the largest number.

Benin is a resource‐limited country, and several advanced diagnostic tests used to better assess the severity of CKD, the adequacy of hemodialysis, and its associated effects—such as iPTH, BUN, nPCR, hs‐CRP, and Kt/V—are not available. We did not assess the association between pruritus and certain parameters such as serum potassium, serum albumin, β2‐microglobulin, blood glucose, and body mass index. These gaps may have limited our ability to demonstrate potential causal relationships with the identified associated factors. Moreover, the cross‐sectional design does not allow for establishing temporality or causality. The factors identified in this study therefore reflect possible associations rather than definitive causal links.

Nevertheless, our study represents a major contribution to understanding pruritus in hemodialysis patients, particularly in individuals with darker skin phototypes living in a resource‐limited setting. By addressing this often overlooked issue, it highlights several risk and protective factors, as well as potential avenues for improving patient management.

## 5. Conclusion

Pruritus is a frequent and debilitating condition among hemodialysis patients. The main risk factors identified in this study were severe xerosis, prolonged duration of hemodialysis, the use of an AVF, and the presence of Beau’s lines, whereas the use of oral antidiabetic medications appeared to be a protective factor.

These findings suggest a potential link in the underlying pathophysiology of the condition. Pending confirmation of causal relationships, this study underscores the need for improved dermatological management and optimization of dialysis practices in order to reduce the frequency of pruritus and enhance patients’ quality of life. It also provides a solid foundation for future multicenter and longitudinal studies aimed at further elucidating pathophysiological mechanisms, optimizing therapeutic strategies, and refining dermatological follow‐up for hemodialysis patients at the national level.

## Author Contributions

All authors contributed to this work.

## Funding

No funding was received for this manuscript.

## Disclosure

All authors have read and approved the final version of the manuscript.

## Conflicts of Interest

The authors declare no conflicts of interest.

## Data Availability

The data that support the findings of this study are available on request from the corresponding author. The data are not publicly available due to privacy or ethical restrictions.
